# Novel Function of α-Cubebenoate Derived from *Schisandra chinensis* as Lipogenesis Inhibitor, Lipolysis Stimulator and Inflammasome Suppressor

**DOI:** 10.3390/molecules25214995

**Published:** 2020-10-28

**Authors:** Su Ji Bae, Ji Eun Kim, Yun Ju Choi, Su Jin Lee, Jeong Eun Gong, Young-Whan Choi, Dae Youn Hwang

**Affiliations:** 1 Department of Biomaterials Science (BK21 Four Program), College of Natural Resources & Life Science/Life and Industry Convergence Research Institute/Laboratory Animal Resources Center, Pusan National University, Miryang 50463, Korea; sujibaebae@pusan.ac.kr (S.J.B.); prettyjiunx@naver.com (J.E.K.); poiu335@naver.com (Y.J.C.); nuit4510@naver.com (S.J.L.); kos93589@naver.com (J.E.G.); 2 Department of Horticultural Bioscience, College of Natural Resources & Life Science, Pusan National University, Miryang 50463, Korea; ywchoi@pusan.ac.kr

**Keywords:** α-cubebenoate, obesity, lipogenesis, lipolysis, inflammasome, cytokines

## Abstract

The efficacy of α-cubebenoate isolated from *Schisandra chinensis* has been previously studied in three disease areas, namely inflammation, sepsis, and allergy, and its role in other diseases is still being explored. To identify the novel function of α-cubebenoate on lipid metabolism and related inflammatory response, alterations in fat accumulation, lipogenesis, lipolysis, and inflammasome activation were measured in 3T3-L1 preadipocytes and primary adipocytes treated with α-cubebenoate. Lipid accumulation significantly decreased in MDI (3-isobutyl-1-methylxanthine, dexamethasone, and insulin)-stimulated 3T3-L1 adipocytes treated with α-cubebenoate without any significant cytotoxicity. The mRNA levels of peroxisome proliferator-activated receptor (PPAR)γ and CCAAT-enhancer binding protein (C/EBP) α for adipogenesis, as well as adipocyte fatty acid binding protein 2 (aP2) and fatty acid synthetase (FAS) for lipogenesis, were reduced after α-cubebenoate treatment, while cell cycle arrest at G2/M stage was restored in the same group. α-cubebenoate treatment induced glycerol release in primary adipocytes and enhanced expression of lipolytic proteins (HSL, perilipin, and ATGL) expression in MDI-stimulated 3T3-L1 adipocytes. Inflammasome activation and downstream cytokines expression were suppressed with α-cubebenoate treatment, but the expression of insulin receptor signaling factors was remarkably increased by α-cubebenoate treatment in MDI-stimulated 3T3-L1 adipocytes. These results indicate that α-cubebenoate may play a novel role as lipogenesis inhibitor, lipolysis stimulator, and inflammasome suppressor in MDI-stimulated 3T3-L1 adipocytes. Our results provide the possibility that α-cubebenoate can be considered as one of the candidates for obesity management.

## 1. Introduction

*Schisandra chinensis* is a plant with significant beneficial effects on various human diseases including cancer, obesity, aging, inflammation, cardiovascular diseases and neurodegenerative disorders based on the function of chemical constituents such as the lignans schisandrin, deoxyschizandrin, gomisins, and pregomisin [1,2]. α-cubebenoate was first identified in the fruits of *S. chinensis* through a series of extraction processes using various solvents including ethyl alcohol (EtOH), chloroform (CHCl_3_), methyl alcohol (MeOH) and hexane [3]. Subsequently, the basic chemical composition and cyclopropane ring system of this compound was determined by ^1^H, ^13^C, distortionless enhancement by polarization transfer (DEPT), heteronuclear single quantum coherence (HSQC), and heteronuclear multiple bond correlation (HMBC) nuclear magnetic resonance (NMR) [3]. A few studies have elucidated the beneficial role of α-cubebenoate in diseases. This molecule significantly inhibited expression of inducible nitric oxide synthase (iNOS) and cyclooxygenase (COX)-2, and production of nitric oxide (NO) and prostaglandin E2 (PGE2) in mouse peritoneal macrophages through suppression of c-Jun N-terminal kinases (JNK) activation [3]. The α-cubebenoate treated cecal ligation and puncture (CLP) experimental model showed enhanced survival, blocked CLP-induced lung inflammation, and increased bactericidal activity. Also, α-cubebenoate treatment resulted in reduction of cytokines such as interleukin (IL)-1β and IL-6 as well as attenuation of apoptosis and caspase-3 activation in lymphocyte [4]. Furthermore, α-cubebenoate suppressed bronchiolar structural changes, accumulation of immune cells and expression of T helper (Th) 2 cytokines induced by ovalbumin challenge in RBL-2H3 mast cells and albino, laboratory-bred strain of the house mice (BALB/c) sensitized with ovalbumin [5]. However, to date, no studies have provided any scientific evidence on the therapeutic effects of α-cubebenoate on lipid metabolism in adipocytes.

Obesity is a chronic condition that promotes the development of metabolic disorders including diabetes, hypertension, stroke, myocardial infarction, insulin resistance and some [6]. Several metabolic targets drugs have been developed to treat obesity. Active constituents and extracts derived from natural products have received attention as potential anti-obesity agents, because of their enhanced efficacy with minimal side effects [7]. The mechanism of action of several compounds on multiple molecular targets leading to anti-obesity effects has been well researched. The mechanisms elucidated for some extracts and active constituents include lipase inhibition, appetite suppression, energy expenditure stimulation, adipocyte differentiation inhibition, and lipolysis activation [6]. Among these targets, adipocyte differentiation was shown to be significantly inhibited by several natural products and active constituents including polyunsaturated fatty acid (PUFA), genistein, capsaicin, ginsenosides, epigallocatechin gallate (EGCG) and sitosterol, while apoptotic effects on maturing pre-adipocytes were induced by esculetin, resveratrol, quercetin, genistein, and capsaicin [6,8,9,10,11,12]. The stimulation of triglyceride hydrolysis is promoted by curcumin extracted from *Curcuma longa* L., licorice flavonoid from *Glycyrrhiza glabra* L., genistein from soybean, and caffeine from *Caffea camephora* [13,14,15,16].

The current study was undertaken to investigate the therapeutic effects and molecular mechanisms of action of α-cubebenoate on lipogenesis, lipolysis, and inflammatory responses in adipocytes. The results of this study provide the first scientific evidence that the anti-obesity effects of α-cubebenoate may be closely associated with inhibition of adipocyte differentiation, stimulation of lipolysis, suppression of inflammasome activation and cytokine expression, and upregulation of insulin receptor signaling molecules in 3T3-L1 adipocytes and primary adipocytes.

## 2. Results

### 2.1. Cytotoxicity of α-Cubebenoate Against 3T3-L1 Adipocytes

**Figure 1:**
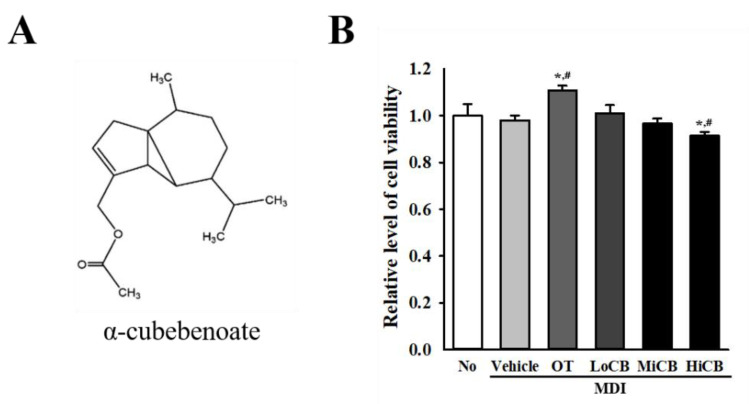
Cytotoxicity. (**A**) Chemical structure of α-cubebenoate. (**B**) Viability of 3T3-L1 adipocytes to α-cubebenoate. After incubation with 10, 20 and 30 µg/mL of α-cubebenoate for 24 h, the cell viability was determined by the MTT assay in duplicate. The data represents the means ± SD of duplicates. * indicates *p* < 0.05 compared to the No (untreated) group. # indicates *p* < 0.05 compared to the MDI+Vehicle treated group. OT; orlistat, MDI; adipogenic cocktail consisting of 3-isobutyl-1-methylxanthine, dexamethasone, and insulin, LoCB; low concentration (10 µg/mL) of α-cubebenoate, MiCB; middle concentration (20 µg/mL) of α-cubebenoate, HiCB; high concentration (30 µg/mL) of α-cubebenoate.

The cytotoxic effect of α-cubebenoate was evaluated against adipocytes. To achieve this, the cell viability of 3T3-L1 adipocytes was determined following treatment with 10 (Low concentration, LoCB), 20 (Middle concentration, MiCB) and 30 (High concentration, HiCB) µg/mL of α-cubebenoate. All treated groups except the MDI+HiCB treated group maintained a constant level of viability when compared to the untreated (No) or Vehicle treated groups. However, a slight decrease in levels of cell viability was observed in the MDI+HiCB treated group, while a reverse pattern was detected in the MDI+orlistat (OT) treated group (Figure 1B). Also, the above results were completely reflected in the cell morphology after α-cubebenoate treatment (data not shown). Therefore, these results suggest that α-cubebenoate does not induce any significant toxicity to 3T3-L1 adipocytes at a concentration less than 20 µg/mL.

### 2.2. Inhibitory Effects of α-Cubebenoate on Lipid Accumulation

**Figure 2:**
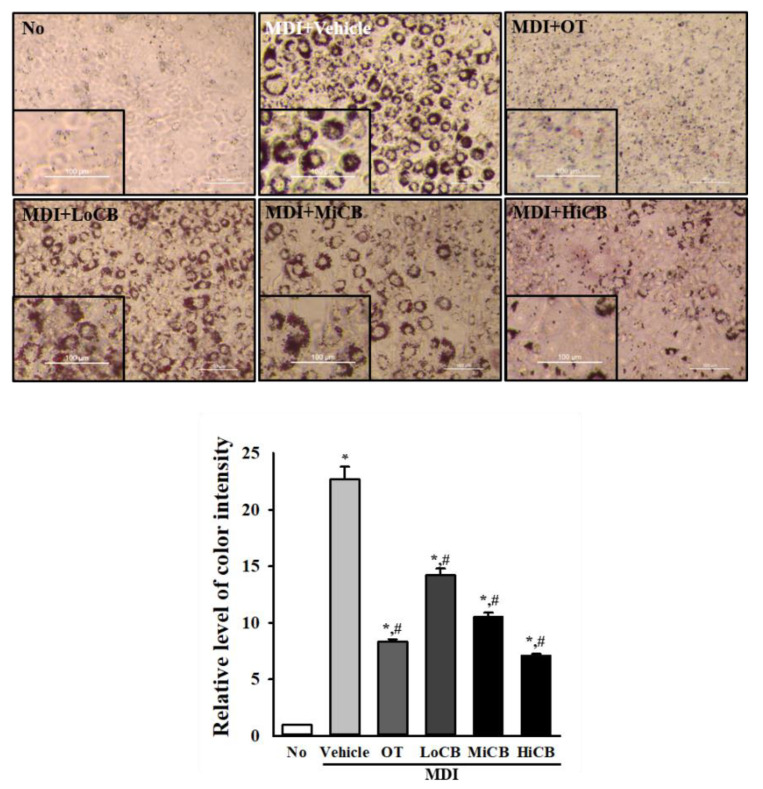
ORO staining analysis. 3T3-L1 pre-adipocytes were cultured in MDI medium with three different concentration of α-cubebenoate for 8 days, after which they were subjected to ORO staining analysis. The images of the ORO stained cells were observed with an inverted microscope at 100× magnification. The level of the stained lipid droplets was quantified by the Image J 1.52a program. The data represents the means ± SD of duplicates. * indicates *p* < 0.05 compared to the No (untreated) group. # indicates *p* < 0.05 compared to the MDI+Vehicle treated group. OT; orlistat, MDI; adipogenic cocktail consisting of 3-isobutyl-1-methylxanthine, dexamethasone, and insulin, LoCB; low concentration (10 µg/mL) of α-cubebenoate, MiCB; middle concentration (20 µg/mL) of α-cubebenoate, HiCB; high concentration (30 µg/mL) of α-cubebenoate.

To examine the inhibitory effects of α-cubebenoate on neutral lipid accumulation, alterations in the levels of Oil Red-O (ORO) stained lipid droplets were measured in differentiated 3T3-L1 adipocytes treated with α-cubebenoate. Oil Red-O-stained materials (ORO-SM) were significantly accumulated in differentiated 3T3-L1 adipocytes (MDI+Vehicle treated group) compared with the No (untreated) group. However, these accumulation levels were remarkably decreased in a dose-dependent manner after treatment with α-cubebenoate. The highest level of their suppression was detected in the MDI+HiCB treated group (Figure 2). These results indicate that α-cubebenoate inhibits the accumulation of neutral lipids in MDI-stimulated 3T3-L1 adipocytes.

### 2.3. Inhibitory Effects of α-Cubebenoate on the Regulation of Adipogenesis and Lipogenesis

**Figure 3:**
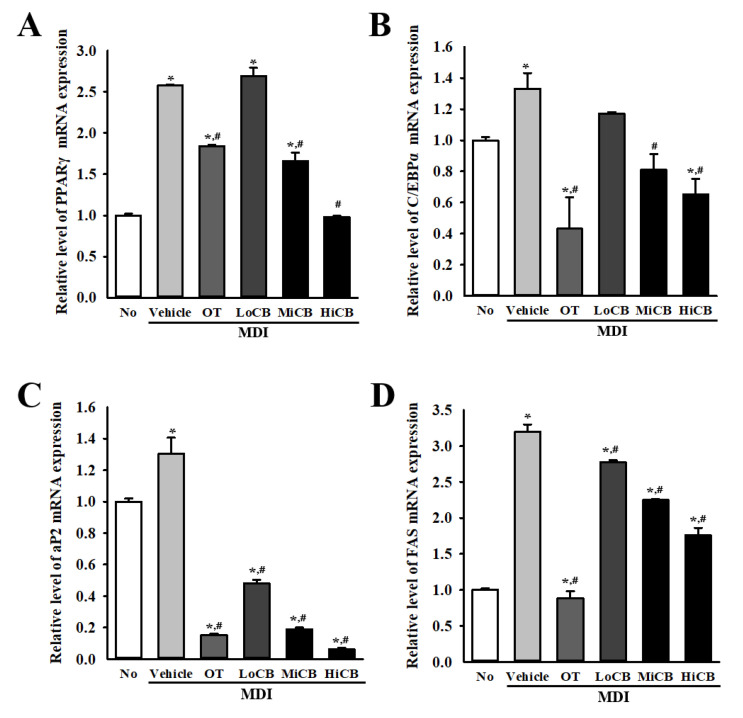
mRNA levels of adipogenic and lipogenic factors. After collection of total RNA from MDI-stimulated 3T3-L1 adipocytes treated with α-cubebenoate, the mRNA levels of two adipogenic transcription factors (PPARγ [**A**] and C/EBPα [**B**]) and two lipogenic proteins (aP2 [**C**] and FAS [**D**]) genes were measured by RT-qPCR as described in materials and methods. The data represents the means ± SD of duplicates. * indicates *p* < 0.05 compared to the No (untreated) group. # indicates *p* < 0.05 compared to the MDI+Vehicle treated group. OT; orlistat, MDI; adipogenic cocktail consisting of 3-isobutyl-1-methylxanthine, dexamethasone, and insulin, LoCB; low concentration (10 µg/mL) of α-cubebenoate, MiCB; middle concentration (20 µg/mL) of α-cubebenoate, HiCB; high concentration (30 µg/mL) of α-cubebenoate.

To investigate whether the inhibitory effect of α-cubebenoate on lipid accumulation is accompanied by suppression of adipogenesis and lipogenesis, alterations in the level of transcription of two adipogenic transcription factors (PPARγ and C/EBPα) and two lipogenic proteins (aP2, FAS) were measured in the differentiated 3T3-L1 adipocytes treated with α-cubebenoate. Similar regulation patterns were observed in the expression levels of four related factors. The mRNA expression of the two transcription factors and the two lipogenic genes was higher in the MDI+Vehicle treated group than in the No (untreated) group, although their rates of increase were variable. However, these levels were significantly decreased in the differentiated 3T3-L1 adipocytes treated with α-cubebenoate compared to the MDI+Vehicle treated group (Figure 3). Specifically, remarkable suppression was observed on the expression levels of aP2 mRNA post α-cubebenoate treatment (Figure 3C). These results suggest that the inhibitory effect of α-cubebenoate on lipid accumulation could be closely linked to the inhibition of transcription of the adipogenic transcription factors (PPARγ and C/EBPα) and the lipogenic proteins (aP2 and FAS).

### 2.4. Regulatory Effects of α-Cubebenoate on the Cell Cycle of 3T3-L1 Adipocyte

**Figure 4:**
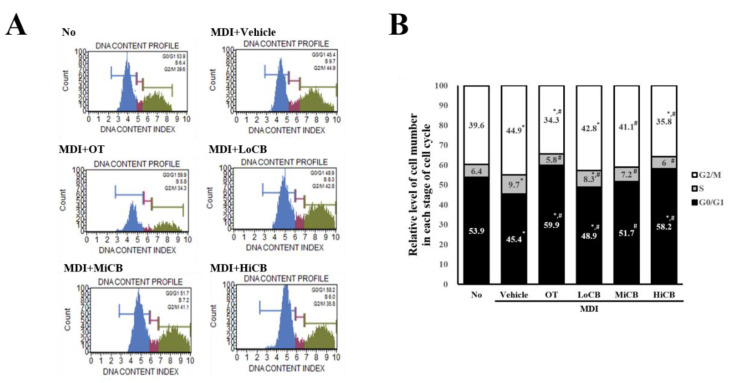
Cell cycle arrest analysis. (**A**) Cell cycle distribution. The cell cycle distribution of MDI-stimulated 3T3-L1 adipocytes treated with α-cubebenoate was analyzed with flow cytometric analysis of the DNA content of nuclei of cells after staining with propidium iodide (PI). (**B**) Analysis of cell number % of each cell cycle phase relative to total phases. The number of cells in the G0/G1, S, and G2/M stage were determined at each time point and each phase % is calculated as the percentage of the number of cells in the specific population relative to the number of total cells. The data represents the means ± SD of three replicates. * indicates *p* < 0.05 compared to the No (untreated) group. # indicates *p* < 0.05 compared to the MDI+Vehicle treated group. OT; orlistat, MDI; adipogenic cocktail consisting of 3-isobutyl-1-methylxanthine, dexamethasone, and insulin, LoCB; low concentration (10 µg/mL) of α-cubebenoate, MiCB; middle concentration (20 µg/mL) of α-cubebenoate, HiCB; high concentration (30 µg/mL) of α-cubebenoate.

To investigate whether inhibition of α-cubebenoate on lipid accumulation and differentiation can affect the regulation of the cell cycle, the number of cells at each stage of the cell cycle was measured in the differentiated 3T3-L1 adipocytes after treatment with α-cubebenoate. During MDI-stimulated differentiation of 3T3-L1 adipocytes, the cell number in the G0/G1 stage was lower than in the No (untreated) group and those in the S and G2/M stage were higher. However, cell numbers in the G0/G1 stage and G2/M stage significantly recovered in a dose-dependent manner in 3T3-L1 adipocytes after treatment with α-cubebenoate (Figure 4). These results suggest that α-cubebenoate treatment restores the cell cycle arrest in the G2/M stage of MDI-stimulated 3T3-L1 adipocytes.

### 2.5. Stimulatory Effects of α-Cubebenoate on the Regulation of Lipolysis

**Figure 5:**
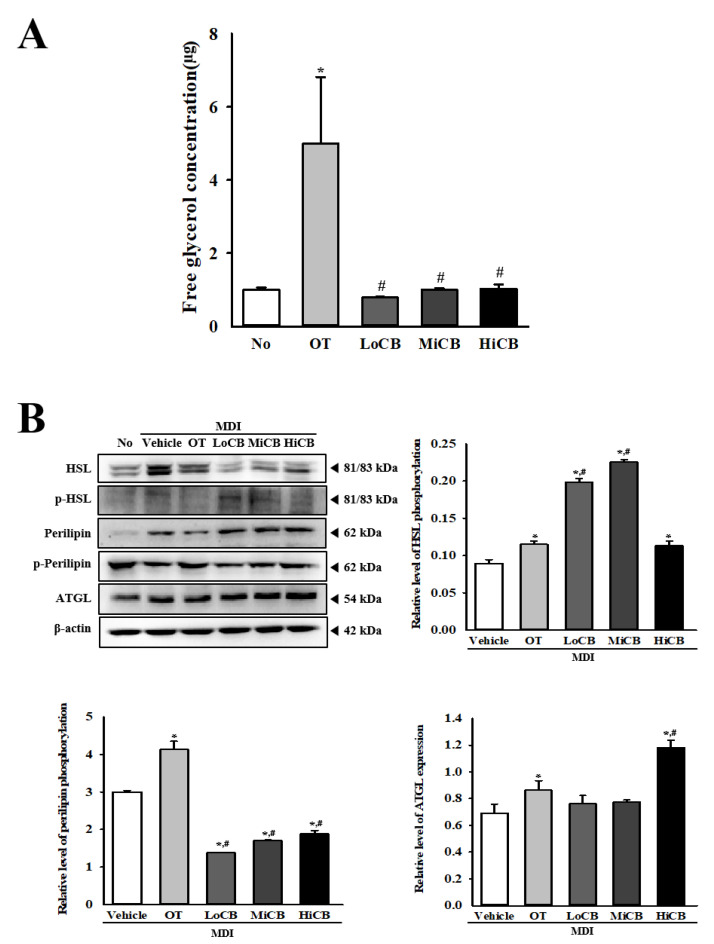
Free glycerol release and lipolytic protein expression. (**A**) Level of free glycerol in culture media. Released glycerol was measured in the supernatant of primary adipocytes treated with three different concentrations of α-cubebenoate. (**B**) Expression level of lipolytic proteins. After the collection of total proteins from MDI-stimulated 3T3-L1 adipocytes treated with α-cubebenoate, the expression levels of HSL, p-HSL, perilipin, p-perilipin, ATGL and β-actin were detected with specific antibodies, followed by horseradish peroxidase-conjugated goat anti-rabbit IgG. Each band intensity was measured using an imaging densitometer, and the relative levels of each protein were calculated relative to the intensity of actin bands. The data represents the means ± SD of three replicates. * indicates *p* < 0.05 compared to the No (untreated) group. # indicates *p* < 0.05 compared to the MDI+Vehicle treated group. OT; orlistat, MDI; adipogenic cocktail consisting of 3-isobutyl-1-methylxanthine, dexamethasone, and insulin, LoCB; low concentration (10 µg/mL) of α-cubebenoate, MiCB; middle concentration (20 µg/mL) of α-cubebenoate, HiCB; high concentration (30 µg/mL) of α-cubebenoate.

We examined the stimulatory effects of α-cubebenoate on the regulation of lipolysis. To achieve this, alteration in the levels of free glycerol and expression levels of lipolytic proteins were determined in the culture medium of primary adipocytes and MDI-stimulated 3T3-L1 cells after treatment with 10, 20, and 30 µg/mL of α-cubebenoate. The concentration of free glycerol in primary adipocyte cultured medium was significantly enhanced in a dose-dependent manner after α-cubebenoate treatment although the OT treated group showed the highest levels (Figure 5A). Also, a similar regulation pattern was observed in the expression of three lipogenic proteins in α-cubebenoate treated MDI-stimulated 3T3-L1 adipocytes. The phosphorylation level of hormone sensitive lipase (HSL) and perilipin were significantly increased in MDI-stimulated 3T3-L1 adipocytes treated with α-cubebenoate although few differences were observed in HSL phosphorylation levels of the MDI+HiCB treated group. The expression levels of adipose triglyceride lipase (ATGL) was similarly increased in all three α-cubebenoate treated MDI-stimulated 3T3-L1 adipocytes (Figure 5B). Overall, these results suggest that α-cubebenoate treatment promotes lipolysis in primary adipocytes derived from Sprague-Dawley (SD) rats and MDI-stimulated 3T3-L1 adipocytes.

### 2.6. Suppression of Inflammasome Activation by α-Cubebenoate

**Figure 6:**
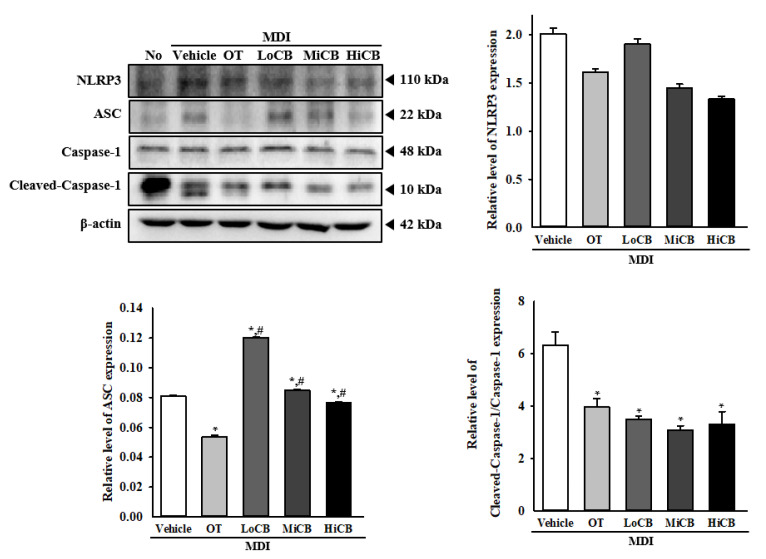
Expression of ASC, caspase-1 and NLRP3 protein. Western blot was performed to detect ASC, caspase-1 and NLRP3 proteins in the homogenates of MDI-stimulated 3T3-L1 adipocytes treated with α-cubebenoate using specific antibodies. After determining the intensity of each band using an imaging densitometer, the relative levels of ASC, caspase-1 and NLRP3 proteins were calculated based on the band intensity of β-actin protein as the endogenous control. The data represents the means ± SD of three replicates. * indicates *p* < 0.05 compared to the No (untreated) group. # indicates *p* < 0.05 compared to the MDI+Vehicle treated group. OT; orlistat, MDI; adipogenic cocktail consisting of 3-isobutyl-1-methylxanthine, dexamethasone, and insulin, LoCB; low concentration (10 µg/mL) of α-cubebenoate, MiCB; middle concentration (20 µg/mL) of α-cubebenoate, HiCB; high concentration (30 µg/mL) of α-cubebenoate.

NLR family pyrin domain containing 3 (NLRP3) inflammasome plays a crucial role in adipose tissues in obesity-associated inflammatory response [17,18]. To investigate the suppressive effects of α-cubebenoate on inflammasome activation, the expression levels of NLRP3, apoptosis-associated speck-like protein containing a CARD (ASC) and caspase-1 were detected in the MDI-stimulated 3T3-L1 adipocytes after α-cubebenoate treatment. The levels of these three proteins were significantly decreased in the α-cubebenoate MDI-stimulated 3T3-L1 adipocytes as compared to the MDI+Vehicle treated group although a constant level of NLRP3 and ASC expression was maintained in the MDI+LoCB treated group. In particular, the relative levels of cleaved caspase-1/caspase-1 were lower in the α-cubebenoate treated group than the OT treated group (Figure 6). These results indicate that α-cubebenoate suppresses inflammasome activation in addition to inhibitory effects on adipogenesis and stimulatory effects on lipolysis.

### 2.7. Suppressive Effects of α-Cubebenoate on the Expression of Inflammasome-Related Cytokines

**Figure 7:**
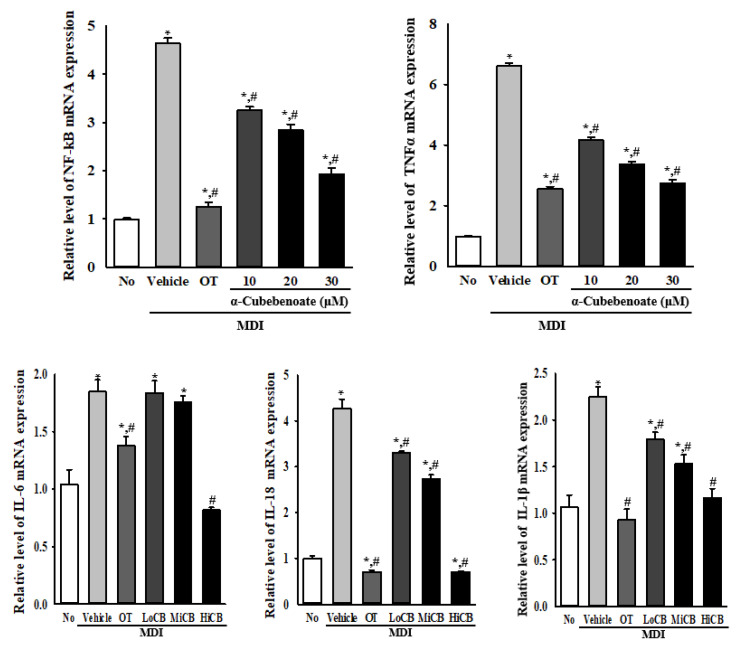
Transcription level of inflammatory cytokines. After collection of total RNA from MDI-stimulated 3T3-L1 adipocytes treated with α-cubebenoate, the mRNA levels of NF-κB, TNFα, IL-6, IL-18 and IL-1β genes were measured by RT-qPCR as described in materials and methods. The data represents the means ± SD of duplicates. * indicates *p* < 0.05 compared to the No (untreated) group. # indicates *p* < 0.05 compared to the MDI+Vehicle treated group. OT; orlistat, MDI; adipogenic cocktail consisting of 3-isobutyl-1-methylxanthine, dexamethasone, and insulin, LoCB; low concentration (10 µg/mL) of α-cubebenoate, MiCB; middle concentration (20 µg/mL) of α-cubebenoate, HiCB; high concentration (30 µg/mL) of α-cubebenoate.

An inflammasome activation can trigger cleavage of caspase-1 and subsequent secretion of some inflammatory cytokines such as IL-1β and IL-18 [19]. To examine whether suppression of inflammasome activation is accompanied by an alteration in the expression of inflammasome-related cytokines, the expression level of several inflammatory cytokines was measured in the differentiated 3T3-L1 adipocytes after treatment with α-cubebenoate. The expression level of nuclear factor-kappa B (NF-κB), tumor necrosis factor (TNF) α, IL-6, IL-18, and IL-1β were remarkably higher in the MDI+Vehicle treated group compared to the No (untreated) group. However, these levels were significantly decreased in MDI-stimulated 3T3-L1 adipocytes treated with α-cubebenoate compared with the MDI+Vehicle treated group. Most of these reductions were α-cubebenoate concentration dependent (Figure 7). These results show that the suppression of inflammasome activation may be closely linked to the inhibition of inflammasome-related cytokine expression in MDI-stimulated 3T3-L1 adipocytes.

### 2.8. Inhibitory Effects of α-Cubebenoate on the Development of Insulin-Resistance

**Figure 8:**
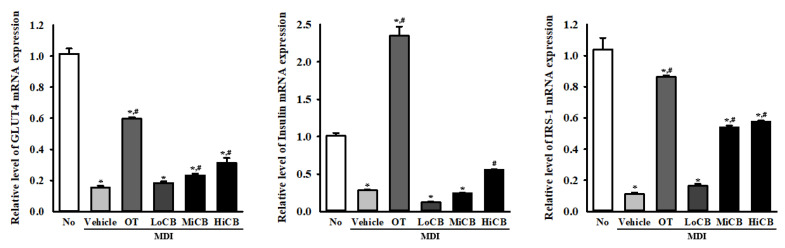
Transcription level of insulin receptor signaling molecules. After collection of total RNA from MDI-stimulated 3T3-L1 adipocytes treated with α-cubebenoate, the mRNA levels of GLUT4, insulin and IRS-1 genes were measured by RT-qPCR as described in materials and methods. The data represents the means ± SD of duplicates. * indicates *p* < 0.05 compared to the No (untreated) group. # indicates *p* < 0.05 compared to the MDI+Vehicle treated group. OT; orlistat, MDI; adipogenic cocktail consisting of 3-isobutyl-1-methylxanthine, dexamethasone, and insulin, LoCB; low concentration (10 µg/mL) of α-cubebenoate, MiCB; middle concentration (20 µg/mL) of α-cubebenoate, HiCB; high concentration (30 µg/mL) of α-cubebenoate.

Inflammasome activation has been implicated in the development of insulin-resistance in adipose tissue of obese patients through impairment of insulin signaling [19]. We investigated whether α-cubebenoate-induced suppression of inflammasome activation is accompanied by an alteration in the expression of insulin receptor signaling molecules. To achieve this, the levels of glucose transporter type (GLUT) 4, insulin and insulin receptor substrate (IRS)-1 transcription were measured in the differentiated 3T3-L1 adipocytes after treatment with α-cubebenoate. A significant suppression of transcription of these factors was observed in the MDI+Vehicle treated group as compared to the No (untreated) group. However, the level of GLUT4, insulin and IRS-1 transcript were remarkably enhanced after treatment with α-cubebenoate although the rate of increase was variable (Figure 8). The above results indicate that α-cubebenoate treatment can induce a recovery of insulin receptor signaling molecule expression through the suppression of inflammasome activation.

## 3. Discussion

α-cubebenoate has received significant attention as a new candidate compound for treatment of certain diseases, including inflammatory disease, sepsis and allergy [3,4,5]. Its therapeutic role in lipid metabolism and related-inflammatory reactions is an important area of research, due to the rising role of obesity in the development of several metabolic diseases. Our study aimed to obtain scientific evidence on the role of α-cubebenoate as a lipogenesis inhibitor, lipolysis stimulator and inflammasome suppressor in 3T3-L1 adipocytes and primary adipocytes. The results of the present study suggest that α-cubebenoate inhibits lipid accumulation in MDI-stimulated 3T3-L1 adipocytes through the suppression of adipogenic transcription factors and stimulation of lipogenic proteins during the early stage of MDI-stimulated adipocytes differentiation. Our results also show that α-cubebenoate inhibits inflammasome activation and downregulates the release of inflammasome-related cytokines as well as upregulates inflammasome-derived insulin receptor signaling.

Furthermore, 3T3-L1 preadipocyte has the ability to differentiate into adipocyte cells and is one of most well-known adipocytes used in anti-obesity drug studies [20,21]. During the differentiation of fibroblast-like preadipocytes into mature lipid accumulated and insulin-responsive adipocytes, two adipogenic transcription factors (PPARγ and C/EBPα) and lipogenic proteins (aP2 and FAS) play an important role [22,23]. The former triggers the expression of adipocyte-specific proteins in the intermediate stage of adipocyte differentiation, while the latter induces the production and maintenance of adipocyte phenotypes in the late stage [21,24]. Therefore, these factors have been widely used as key markers to identify novel compounds with anti-obesity activity. In 3T3-L1 preadipocytes, the levels of these four markers were significantly enhanced after differentiation [25,26,27]. Several single compounds such as eupatilin [28], resveratrol [29], zeaxanthin [30] and ramalin [31] are known to effectively inhibit the expression of these four markers four markers in MDI-stimulated differentiated adipocytes although the compounds displayed variable levels of inhibition. In this study, we measured the levels of the four markers to verify the inhibitory effect of α-cubebenoate on adipogenesis and lipogenesis in MDI-stimulated 3T3-L1 adipocytes. α-cubebenoate inhibited the expression of two adipogenic transcription factors and lipogenic proteins in our study. Similar results have been reported in previous studies. Therefore, these results provide scientific evidence to suggest that α-cubebenoate can act as a novel adipogenesis and lipogenesis inhibitor.

Further, in this study, the stimulatory activity of α-cubebenoate on lipolysis was determined using glycerol assay in primary adipocytes and western blot in MDI-stimulated 3T3-L1 adipocytes. As shown in Figure 5, α-cubebenoate treatment had a significant stimulatory effect on lipolysis, although the rate of increase was different with each factor. However, lipolytic effects of a single compound with anti-obesity activity has not been determined in earlier studies. Treatment with resveratrol has been shown to reduce glycerol release and perilipin expression in TNF-α-stimulated 3T3-L1 adipocytes, while a similar effect was detected in MDI-stimulated 3T3-L1 cells treated with morusin [29,32]. Also, the expression level of perilipin mRNA was suppressed with zeaxanthin treatment in MDI-stimulated 3T3-L1 cells [30]. The results of these single compounds in MDI-stimulated 3T3-L1 adipocytes were similar to those seen in our study. Therefore, the results of our study appear to suggest that α-cubebenoate may have a specific function as a lipolysis stimulator. However, our study has been carried out through limited analyses of glycerol release only in primary adipocytes and lipolytic proteins expression only in MDI-stimulated 3T3-L1 adipocytes.

Inflammasomes are formed as a molecular platform when the activation of NLRP3 and HIN-200 protein, absent in melanoma 2 (AIM2), ASC and pro-caspase-1 during the cellular stress response. These complexes stimulate autoactivation of caspase-1 and subsequently induce the activation of mediators of inflammation and immune response, including IL-1β and IL-18 [19]. During this activation, IL-1β triggers the secretion of IL-6 and TNF-α to regulate cell migration and infiltration, while IL-18 induces the recruitment and activation of immune cells [33]. Meanwhile, some compounds specifically inhibit activation of NLRP3 inflammasome and cytokine production in adipocytes although NLRP3 inflammasomes were activated in obesity-induced inflammation [17,18]. Sodium butyrate (NaB) inhibits the activation of NLRP3 inflammasome and cytokine expression in 3T3-L1 adipocytes pretreated with TNF-α to mimic the inflammatory state [34]. Hydrogen sulfide (H2S) inhibits the expression of inflammasome regulators, and the production of IL-1β and IL-18 in 3T3-L1 adipocytes during high glucose (HG)-induced NLRP3 inflammasome activation [35]. Thus, the results of the current study are consistent with previous studies wherein we detected a decrease in the expression of three inflammasome regulators and five cytokine transcriptions in MDI-stimulated 3T3-L1 adipocytes after α-cubebenoate treatment. Our results primarily provide scientific evidence for the molecular mechanism of the anti-inflammatory effect of α-cubebenoate on inflammatory response in MDI-stimulated of 3T3-L1 adipocytes.

## 4. Materials and Methods

### 4.1. Purification of α-Cubebenoate

α-Cubebenoate (Figure 1A) was isolated and purified as described in a previous study [3]. Briefly, the fruits of *S. chinensis* (Turcz.) Baill were collected in September 2010 in Moonkyong, South Korea and identified by Professor Young Whan Choi, Department of Horticultural Bioscience, Pusan National University. A voucher specimen (accession no. SC-PDRL-2) has been deposited in the Herbarium at the Pusan National University.

**Figure 9:**
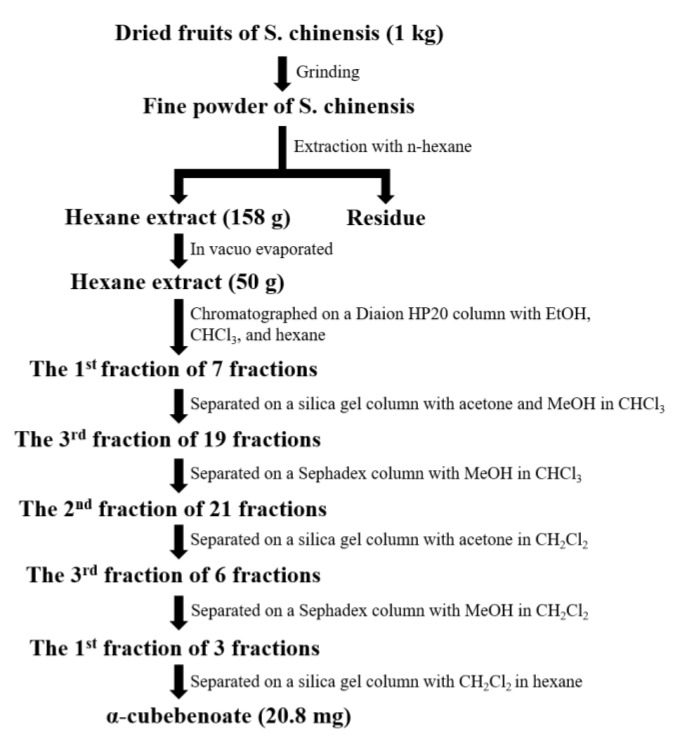
Schematic procedure of α-cubebenoate preparation. After preparation of fine powder of *S.chinensis*, α-cubebenoate was extracted through six times of chromatography using EtOH, CHCl_3_, MeOH, and CH_2_Cl_2_ as described in the Materials and Methods.

To isolate α-cubebenoate, the dried fruits of *S. chinensis* (1.0 kg) were ground to a fine powder. The active ingredient was extracted successively with n-hexane (3 L) at room temperature. The hexane extract (158 g) was in vacuo evaporated and 50 g was chromatographed on a Diaion HP20 (250–850 μm; Sigma-Aldrich, St. Louis, MO, USA) column (40 × 8 cm^2^) using EtOH, chloroform, and hexane to obtain 7 fractions. The first fraction (17SCH1, 22.95 g) was separated on a silica gel column (80 × 6.5 cm^2^) sequentially with 0.5% acetone, 25% acetone, and 25% MeOH in CHCl_3_ to obtain 19 fractions. The 3rd fraction (17SCH1IC, 430.2 mg) was separated on a Sephadex column (100 × 3.0 cm^2^) using 50% MeOH in CHCl_3_ to obtain 21 fractions, and the 2nd fraction (17SCH1ICIB, 161.9 mg) was separated on a silica gel column (60 × 2.0 cm^2^) using 5% acetone in CH_2_Cl_2_ to obtain 6 fractions. The 3rd fraction (17SCH1ICIBIC, 130.4 mg) was then separated on a Sephadex column (100 × 3.0 cm^2^) using 50% MeOH in CH_2_Cl_2_ to obtain 3 fractions. Finally, the first fraction (17SCH1ICIBICIA, 101.9 mg) was separated on a silica gel column (73 × 3.0 cm^2^) using 50% CH_2_Cl_2_ in hexane to yield α-cubebenoate (17SCH1ICIAIL, 20.8 mg) (Figure 9).

### 4.2. Cell Culture and Adipocyte Differentiation

It is known that 3T3-L1 preadipocytes have the potential to differentiate into adipocyte-like phenotypes. The cells were obtained from the American Type Culture Collection (Mannassas, VA, USA). Cells were cultured in Dulbecco Modified Eagle’s Medium (DMEM, Welgene, Gyeongsan-si, Korea) supplemented with 10% fetal bovine serum (FBS, Welgene), L-glutamine, penicillin, and streptomycin (Thermo Scientific, Waltham, MA, USA), in a humidified incubator at 37 °C under 5% CO_2_ and 95% fresh air. Differentiation of 3T3-L1 preadipocytes was induced following a previously described method [36]. Briefly, cells were grown to more than 80–90% confluence (differentiation day 0). Normal media was then replaced with differentiation medium (MDI) containing 3-isobutyl-1-methylxanthine (0.5 mM, Sigma-Aldrich Co.), dexamethasone (1 µM, Sigma-Aldrich Co.) and insulin (5 µg/mL, Sigma-Aldrich Co.) in DMEM supplemented with 10% fetal bovine serum (FBS). After two days (differentiation day 2), cells were maintained in DMEM supplemented with 10% FBS and 5 µg/mL insulin for two more days (differentiation day 4), followed by culturing for an additional four days in DMEM supplemented with 10% FBS (differentiation day 8). Finally, α-cubebenoate was added to the medium at three different concentrations (10, 20, and 30 µg/mL in dimethyl sulfoxide (DMSO) solution, Duchefa Biochemie, Haarlem, Netherlands) throughout the entire culture period (differentiation day 0 to day 8).

### 4.3. Cell Viability Assay

Cell viability was determined using the tetrazolium compound 3-[4,5-dimethylthiazol-2-yl]-2,5diphenyltetrazolium bromide (MTT) assay (Sigma-Aldrich Co.). To determine the cell viability, 3T3-L1 adipocytes were seeded at a density of 1 × 10^4^ cells/0.2 mL and grown for 24 h in a 37 °C incubator. When the cells attained 70–80% confluence, they were treated with Vehicle (DMSO, a solvent for melting α-cubebenoate), OT (40 µg/mL, Sigma-Aldrich Co.), 10 µg/mL of α-cubebenoate (LoCB), 20 µg/mL of α-cubebenoate (MiCB), or 30 µg/mL of α-cubebenoate (HiCB). Following incubation for 24 h, the supernatants of the 3T3-L1 adipocytes were discarded, after which 0.2 mL of fresh DMEM media and 50 µL of MTT solution (2 mg/mL in PBS) were added to each well. Cells were then incubated at 37 °C for 4 h, after which the formazan precipitate was dissolved in DMSO and the absorbance was read at 570 nm using a VERSA max Plate reader (Molecular Devices, Sunnyvale, CA, USA).

### 4.4. ORO Staining

Lipid accumulation was detected in 3T3-L1 adipocytes after staining with ORO dye, as described in previous reports [37]. Briefly, adipocytes of the subset group were fixed with 4% formaldehyde for 60 min and washed three times with distilled water, after which they were incubated with 0.5% ORO dye (Sigma-Aldrich Co.) in 100% isopropanol (Sigma-Aldrich Co.) for 30 min at room temperature. After washing three times with distilled water, the stained fat droplets in the adipocytes were observed microscopically at 100× magnification (Leica Microsystems, Wetzlar, Germany). The color intensity of stained lipid droplets was measured under the Image J 1.52a program (NIH, Bethesda, ML, USA).

### 4.5. Isolation and Culture of Primary Adipocytes from SD Rats

The protocol for animal experiments was reviewed and approved by the Pusan National University Institutional Animal Care and Use Committee (PNU-IACUC; Approval Number PNU-2017-1461). All Sprague-Dawley (SD) rats were handled at the Pusan National University-Laboratory Animal Resources Center, which is accredited by the Korea Food and Drug Administration (FDA) (Accredited Unit Number-000231) and AAALAC International (Accredited Unit Number; 001525). Eight-week-old male SD rats were purchased from Samtako Bio Korea Inc. (Osan, Korea) and provided with *ad libitum* access to water and a standard irradiated chow diet (Samtako Bio Korea Inc.). During the experiment, rats were maintained in a specific pathogen-free state (SPF) under a strict light cycle (lights on at 08:00 and off at 20:00) at 23 ± 2 °C and 50 ± 10% relative humidity.

To isolate primary adipocytes from adipose tissue of rats, the intra-abdominal adipose tissues were collected from the adult SD rats. This tissue (30 g) was minced in 5 mL of DMEM supplemented with 1 mg/mL type I collagenase (Worthington Biochemical Co., Freehold, NJ, USA) and 1% bovine serum albumin (BSA) (MP Biomedicals, Illkirch, France), and subsequently incubated at 37 °C for 30 min in a shaking incubator (JSR, Gongju-City, Korea). The homogenate of the minced adipose tissue was filtered through a 100 µm nylon mesh and washed three times in KRBH (Krebs ringer/HEPES solution: 25 mM NaHCO_3_, 125 mM NaCl, 5 mM glucose, 2.5 mM KCl, 1.25 mM NaH_2_PO_4_, 2 mM CaCl_2_, 1 mM MgCl_2_, 25 mM HEPES) containing 1% BSA. Finally, after centrifugation, the pellets of the harvested adipocytes were resuspended in KRBH containing 3% BSA. Primary adipocytes were cultured in KRBH supplemented with 3% BSA and maintained in a humidified incubator at 37 °C under 5% CO_2_ and 95% air. Thereafter, the adipocytes were seeded onto 24-well plates for each experimental protocol and incubated with different concentrations of α-cubebenoate to measure the release of free glycerol.

### 4.6. Quantitative Reverse Transcription Polymerase Chain Reaction (RT-qPCR) Analysis

The mRNA levels of PPARγ, C/EBPα, aP2, and FAS were measured by RT-qPCR as previously described [38]. Briefly, total RNA molecules were purified from the cultured cells using RNAzol (Tel-Test Inc., Friendswood, TX, USA). After quantification of RNA using a NanoDrop system (Biospecnano, Shimadzu Biotech, Kyoto, Japan), the complement DNA (cDNA) was synthesized using a mixture of total RNA (5 µg), oligo-dT primer (Invitrogen, Carlsbad, CA, USA), dNTP and reverse transcriptase (Superscript II, 18064-014, Invitrogen, 200 U/µL). qPCR was conducted with a cDNA template and 2× Power SYBR Green (Toyobo Co., Osaka, Japan) using the following cycles: 15 s at 95 °C, 30 s at 55 °C, and 60 s at 70 °C. The primer sequences for target gene expression identification were as follows: PPARγ, sense primer: 5′-GAG TTC ATG CTT GTG AAG GAT GCA AGG-3′, anti-sense primer: 5′-CAT ACT CTG TGA TCT CTT GCA CG-3′; C/EBPα, sense primer: 5′-GTG GAC AAG AAC AGC AAC GAG TAC-3′, anti-sense primer: 5′-GGA ATC TCC TAG TCC TGG CTT GC-3′; FAS, sense primer: 5′-GAT CCT GGA ACG AGA ACA CGA TCT GG-3′; anti-sense primer: 5′-AGA CTG TGG AAC ACG GTG GTG GAA CC-3′; aP2, sense primer: 5′-GAA CCT GGA AGC TTG TCT CCA GTG-3′; anti-sense primer: 5′-GAT GCT CTT CAC CTT CCT GTC GTC TGC-3′; GLUT4, sense primer: 5′-CTA GCT GAG CTG AAG GAT GAG AAA C -3′, anti-sense primer: 5′-GTC GTC CAG CTC GTT CTA CTA AGA G-3′; Insulin, sense primer: 5′-ACC TGG TAG AGG CTC TCT ACC TGG TGT G-3′, anti-sense primer: 5′-GTT GCA GTA GTT CTC CAG CTG GTA GAG-3′; IRS-1, sense primer: 5′-GAG ATC TCG AAC TGA GAG CAT CAC TGC-3′, anti-sense primer: 5′-CAC TGG TAC TAC TAG ATG ACA GAC TC-3′; NF-κB, sense primer: 5′-GTA AC A GCA GGA CCC AAG GA-3′, anti-sense primer: 5′-AGC CCC TAA TAC ACG CCT CT-3′; TNF-α, sense primer: 5′-CCT GTA GCC CAC GTC GTA GC-3′, anti-sense primer: 5′-TTG ACC TCA GCG CTG ACT TG-3′; IL-6, sense primer: 5′-CTC TCT GCA AGA GAC TTC CAT CCA G -3′, anti-sense primer: 5′-GCT ATG GTA CTC CAG AAG ACC AGA GG-3′; IL-18, sense primer: 5′-GTA CAA AGA CAG TGA AGT AAG AGG ACT G-3′, anti-sense primer: 5′-CTC CAT CTT GTT GTG TCC TGG AAC ACG-3′; IL-1β, sense primer: 5′-CTG TCC TGA TGA GAG CAT CCA GCT TC-3′, anti-sense primer: 5′-GTT GCT TGG TTC TTC TTG TAC AAA GCT C-3′; β-actin, sense primer: 5′-TGG AAT CCT GTG GCA TCC ATG AAA C-3′, anti-sense primer: 5′-TAA AAC GCA GCT CAG TAA CAG TCC G-3′. The reaction cycle during which PCR products exceeded this fluorescence intensity threshold during the exponential phase of the PCR amplification was considered the threshold cycle (Ct). Following Livak and Schmittgen’s method, the expression of the target gene was quantified relative to that of the housekeeping gene β-actin, based on a comparison of the Cts at a constant fluorescence intensity [39].

### 4.7. Western Blot Analysis

For the Western blot assay, total protein of 3T3-L1 adipocytes was extracted using the Pro-Prep Protein Extraction Solution (iNtRON Biotechnology, Seongnam, Korea), followed by quantification using a SMARTTM BCA Protein Assay Kit (Thermo Scientific). Equal amounts of proteins (30 µg) were loaded and separated by 4–20% sodium dodecyl sulfate–polyacrylamide gel electrophoresis (SDS-PAGE) for 2 h, after which the resolved proteins were transferred to nitrocellulose membranes for 2 h at 40 V. Each membrane was then incubated separately overnight at 4 °C with the following primary antibodies, all procured from Cell Signaling Technology (Danvers, MA, USA) and diluted 1:1000: anti-perilipin antibody (Cell Signaling Technology), anti-p-perilipin antibody (Cell Signaling Technology), anti-HSL antibody (Cell Signaling Technology), anti-p-HSL antibody (Cell Signaling Technology), anti-ATGL antibody (Cell Signaling Technology), anti-NLRP3 antibody (Cell Signaling Technology), anti-ASC antibody (Cell Signaling Technology), anti-Caspase-1 antibody (Cell Signaling Technology), and anti-β-actin antibodies (Cell Signaling Technology). The probed membranes were then washed with washing buffer (137 mM NaCl, 2.7 mM KCl, 10 mM Na_2_HPO_4_, and 0.05% Tween 20) and incubated with 1:1000 diluted horseradish peroxidase (HRP)-conjugated goat anti-rabbit IgG (Invitrogen) at room temperature for 1 h. Finally, the membrane blots were developed using Amersham ECL Select Western Blotting detection reagent (GE Healthcare, Little Chalfont, UK). The chemiluminescence signals that originated from specific bands were detected using FluorChemi^®^FC2 (Alpha Innotech Co., San Leandro, CA, USA).

### 4.8. Cell Cycle Assay

The cell cycle of 3T3-L1 adipocytes was evaluated using a Muse™Cell Cycle Kit (MCH100106, Millipore Co., Billerica, MA, USA) according to the manufacturer’s instructions. Briefly, 3T3-L1 adipocytes were cultured in 100 mm^2^ dishes (3 × 10^5^ cells/dish), then treated with MDI and three different concentrations of α-cubebenoate (10, 20, and 30 µg/mL) for 24 h. Total cells from subset groups were harvested by centrifugation at 3000× *g* for 5 min and fixed with 70% EtOH at 20 °C for 3 h. The fixed cells were washed with 1× PBS and resuspended in 200 µL of cell cycle reagent. Following incubation at 37 °C in a CO_2_ incubator for 30 min, cell cycles were analyzed using FACS (Millipore Co.).

### 4.9. Measurement of Free Glycerol Release

Free glycerol release from primary adipocytes was measured using the free glycerol reagent (Sigma-Aldrich Co.) as described in a previous study [32]. To measure the glycerol level, primary adipocytes were seeded at a density of 2 × 10^5^ cells/mL in KRBH and cultured in a 37 °C incubator. After 1 h, they were either No (untreated), treated with Vehicle (DMSO), or pretreated with 40 µg/mL of OT or 10, 20, and 30 µg/mL of α-cubebenoate. Following incubation for 24 h, the culture medium was collected from the primary adipocytes, treated with α-cubebenoate and heated at 65 °C for 15 min to inactivate any enzymes released by the adipocytes. The inactivated medium (10 µL) was then mixed with 200 µL of glycerol detection reagent, after which the absorbance was read at 540 nm using a Vmax plate reader (Molecular Devices).

### 4.10. Statistical Significance Analysis

Statistical significance was evaluated using a one-way analysis of variance (ANOVA) (SPSS for Windows, Release 10.10, Standard Version, Chicago, IL, USA) followed by Tukey’s post hoc t-test for multiple comparison. All data were expressed as the means ± SD. A *p* value less than 0.05 was considered statistically significant.

## 5. Conclusions

In the present study, we identified the novel function and molecular mechanism of action of α-cubebenoate on lipogenesis, lipolysis, and inflammatory response in adipocytes. Our results provide scientific evidence that α-cubebenoate inhibits lipogenesis through its effects on the expression of adipogenic factors and cell cycle arrest, while it stimulates lipolysis via the regulation of major lipid droplet-associated proteins in differentiated adipocytes. The results further suggest that α-cubebenoate suppresses inflammasome activation and inflammatory cytokine expression in the same cells. However, additional studies for analyses of molecular mechanisms in vivo in obese animal models are needed to clarify the role of α-cubebenoate as a lipogenesis inhibitor, lipolysis stimulator, and inflammasome activation inhibitor. In addition, some of the important problems such as extraction yield, purity and economics will be encountered during the large-scale production and purification of α-cubebenoate.
